# Root-Zone Heat Priming Effects on Maximum Quantum Efficiency of PSII, Productivity, Root Morphology and Nutritional Quality of Two Aeroponically Grown Leafy Greens in a Tropical Greenhouse

**DOI:** 10.3390/plants11131684

**Published:** 2022-06-25

**Authors:** Jie He, Cassander Tan, Lin Qin

**Affiliations:** Natural Sciences and Science Education Academic Group, National Institute of Education, Nanyang Technological University, 1 Nanyang Walk, Singapore 637616, Singapore; cassandertan@gmail.com (C.T.); lin.qin@nie.edu.sg (L.Q.)

**Keywords:** root-zone heat priming, leafy greens, maximum quantum efficiency of PSII, nutritional quality, productivity, root morphology

## Abstract

This study investigated the effects of root zone (RZ) heat priming on *Eruca sativa* (cv. Arugula) and *Lactuca* *sativa* (cv. Canasta) in a tropical greenhouse by exposing them to three different RZ temperatures (RZTs). For the first group, plants were grown at 25 °C-RZT for 36 days. The second group of plants was grown at 25 °C-RZT for 10 days before transferring to 38 °C-RZT for 10 days and then to 42 °C-RZT for another 16 days (primed). For the third group, plants were grown at 25 °C-RZT for 20 days and for the last 16 days were transferred to 42 °C-RZT (non-primed). RZ heat priming did not affect the leaf expansion of Arugula while the leaf expansion was slower in RZ heat primed Canasta compared to 25 °C-RZT plants. After transferring to 42 °C-RZT in the later stage, RZ heat primed Arugula had similar productivity but a larger root system with higher nutritional quality at harvest and during postharvest storage compared to those of 25 °C-RZT plants. After subjection to 42 °C-RZT, although RZ heat priming enhanced its nutritional quality, the productivity of Canasta was compromised. The results of this study suggest that it is feasible to enhance productivity and nutritional quality of leafy greens at lower production cost through RZT management.

## 1. Introduction

Heat stress caused by global warming might lead to a reduction in crop productivity, including vegetable crops [1,2,3]. In tropical climates, heat stress may become a major limiting factor in field crop production and ultimately, the food security [4]. High temperatures alter membrane stability, thus affecting the activity of chloroplast-based enzymes. It has been reported that photosynthetic functions of plants are highly sensitive to high temperature, where heat stress could impair photosynthetic performance [4,5]. Plants, being sessile organisms that cannot escape heat, are highly vulnerable to high temperatures [6]. Therefore, in light of interminable adverse conditions, acclimatization strategies are required to enhance plants’ tolerance to heat stress in order to sustain crop yield [3,7].

One such strategy is known as heat priming, where plants are subjected to a moderate level of heat stress, followed by an exposure to an abrupt episode of severe heat stress, which has been reported to have enhanced plants’ survivability at lethal temperatures [7,8]. Plants are able to acquire thermotolerance by subjecting them to a sublethal high temperature (“hardening” or “priming”). Heat priming protects plants from a subsequent lethal heat stress. Tolerance to lethal high temperatures can develop more rapidly, within a few hours [9]. The ability of plants to tolerate high temperature without heat priming is defined as basal thermotolerance. Acquired thermotolerance refers to the capacity to tolerate high temperatures after heat priming [10]. The pre-exposure of a plant to sublethal high temperature could trigger a plant’s “stress memory”, helping it to better prepare itself for subsequent stress events [11]. For instance, heat priming applied to wheat with 8 °C (day/night temperature) higher compared to control for 2 days, resulted in enhanced tolerance to high temperature stress occurring after anthesis. Under post-anthesis high temperature stress, heat primed plants showed higher photosynthetic capacity than in the non-primed plants [12]. Thus, the plant that has undergone moderate heat stress is primed to withstand high temperatures that are considered fatal to non-primed plants [13].

High temperature generated in the tropical greenhouse reduces photosynthetic performance, plant growth and productivity of subtropical and temperate vegetable crops. As roots are more thermosensitive than shoots [14], our research team has successfully grown subtropical and temperate crops in a tropical greenhouse through cooling only their roots using an innovative aeroponic farming system [15,16,17,18,19]. Various crops, including temperate vegetables [19] were aeroponically grown in Singapore throughout the year by cooling only their root-zone (RZ) while the aerial parts were subjected to hot ambient temperature. On bright sunny days, temperature inside the tropical greenhouse could be as high as 42 °C. However, temperate lettuce has been successfully grown in aeroponic systems by maintaining its roots around 25 °C while their aerial parts were exposed to hot ambient temperature in the greenhouse [15,16]. In our further study with temperate lettuce, it was found that 20 °C-RZT lettuce plants had much higher maximum quantum efficiency of PSII measured by midday F_v_/F_m_ ratio than that of hot ambient RZT (A-RZT) plants on both sunny and cloudy days [17]. Similar results were found in subtropical vegetable Chinese broccoli [18]. However, the high energy cost was still incurred, even when just cooling only the RZ of the vegetable crops. Screening thermotolerant crops would be one of the means to minimize the production cost. Thus, heat priming could serve as a promising mean to reduce high energy cost incurred in the growing of temperate vegetable crops in the tropical greenhouse.

Plant growth and development, membrane stability, photosynthesis and water relations are adversely affected by high temperature [20,21]. Although there are reports on priming of seeds, cell lines and the entire plants, very little information is available on the RZ heat priming effects on photosynthetic performances, productivity and root morphology. Our previous study showed that RZ heat priming at sublethal RZT of 38 °C did not have any negative impact on root morphology for two different leafy greens, namely, *Eruca sativa* (cv. Arugula) and *Lactuca sativa* (cv. Canasta) over a 10-day priming period [22].

Temperature is considered as an important physical factor that affects not only plant growth but also production of secondary metabolites [11,12,23,24]. It was reported that increasing temperature might enhance the accumulation of all secondary metabolites in plants [24]. It has also been reported that high temperature treatment in the range of 39 °C to 42 °C is beneficial to maintain the quality and postharvest quality of vegetables [25,26]. According to Arah et al. [27], postharvest management quality of fruit starts from harvesting until reaching the final consumer. However, the effects of heat priming at the RZ on quality and postharvest quality of vegetable species grown in the tropical greenhouse are poorly understood. Thus, using two temperate leafy greens, Arugula and Canasta, this study aimed to (1) investigate the impacts of RZ heat priming on photosynthetic performance, productivity and root morphology and (2) evaluate the effects of RZ heat priming on nutritional quality at harvest and during postharvest storage. We hypothesized that RZ heat priming of temperate leafy greens at a sublethal high temperature would enhance photosynthetic performance and productivity as well as nutritional quality after subjecting them to a subsequent high RZT. To test our hypothesis, both Arugula and Canasta plants were grown in a tropical greenhouse under ambient conditions but their roots were exposed to three different conditions: (1) 25 °C-RZT (defined as optimal RZT); (2) 25 °C-RZT → 38 °C-RZT → 42 °C-RZT (defined as RZ heat priming) and (3) 25 °C-RZT → 42 °C-RZT (defined as non-priming). Details of different RZT treatments can be found in Section 4.2. The findings of the study will enhance our understanding through RZ heat priming to enhance thermotolerance of leaf vegetables that could improve crop productivity, quality and postharvest quality at lower production cost.

## 2. Results

### 2.1. Maximum Quantum Efficiency of PSII Measured by Midday Chlorophyll (Chl) Fluorescence F_v_/F_m_ Ratio and Leaf Expansion during RZ Heat Priming

**Figure 1:**
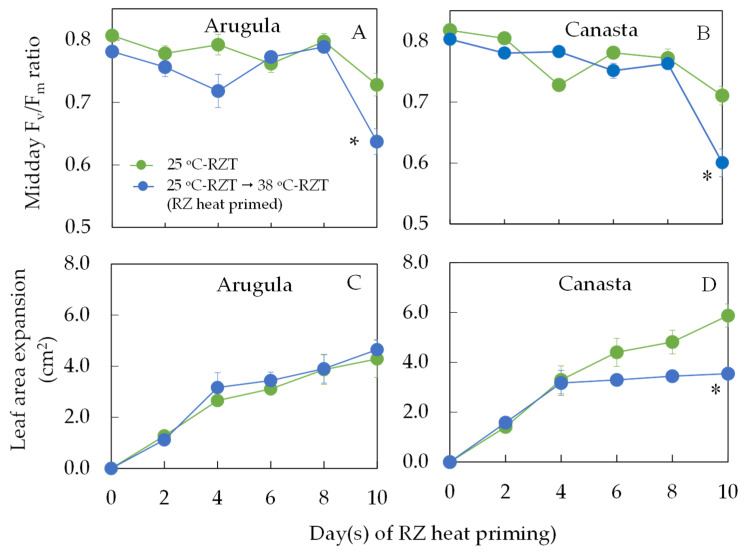
Changes of midday F_v_/F_m_ ratio (**A**,**B**) and leaf expansion (**C**,**D**) of Arugula (**A**,**C**) and Canasta (**B**,**D**) during RZ heat priming period. Vertical bars represent the standard errors (*n* = 4). Asterisk * denotes significant difference between treatment group by one-way ANOVA. Each species was analyzed separately; 25 °C-RZT: plants were grown at a constant 25 °C-RZT for 24 h daily; 25 °C-RZT → 38 °C-RZT: plants transferred from 25 °C-RZT to fluctuating A-RZT with a constant 38 °C-RZT maintained from 1100 to 1700 h for 6 h daily.

During the RZ heat priming period from 10 to 20 DAT, changes in midday F_v_/F_m_ ratio and leaf area expansion were analyzed. After RZ heat priming for 10 days, for both species, midday F_v_/F_m_ ratio of RZ heat primed plants were significantly lower compared to those of 25 °C-RZT plants (Figure 1A,B). No significant difference in leaf area expansion for Arugula between 25 °C-RZT plants and RZ heat primed plants was observed (Figure 1C). However, for Canasta, leaf area expansion was faster in 25 °C-RZT than in RZ heat primed plants after RZ heat priming for 10 days (Figure 1D).

### 2.2. Effects of High RZT on Maximum Quantum Efficiency of PSII of RZ Heat Primed and Non-Primed Plants

**Figure 2:**
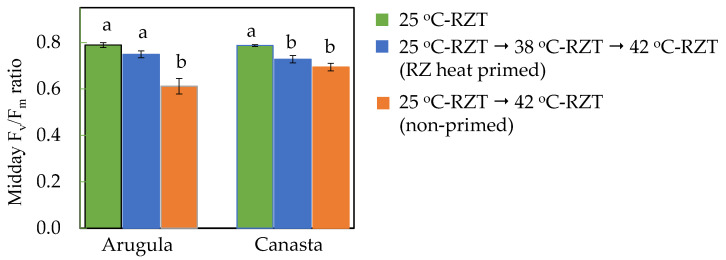
Midday F_v_/F_m_ ratios measured on 33 DAT. The vertical lines through the means represent standard errors (*n* = 4). Different lowercase letters are statistically different (*p* < 0.05, Tukey’s multiple comparison test). Each species was analyzed separately. Refer to Section 4.2 for further details on different RZT treatments.

After exposing both RZ heat primed and non-primed plants to fluctuating A-RZT but with a constant high RZT of 42 °C during midday for 6 h daily, maximum quantum efficiency of PSII measured by midday F_v_/F_m_ ratios were monitored 3 days prior to harvest (33 DAT) and the results are shown in Figure 2. For both species, non-primed plants had the lowest F_v_/F_m_ ratios compared to those of 25 °C-RZT plants and RZ heat primed plants. Although RZ heat primed Arugula plants had a slightly lower F_v_/F_m_ ratio compared to those grown in 25 °C-RZT, the differences were not significant. However, compared to those of 25 °C-RZT Canasta, both RZ heat primed and non-primed plants had significantly lower F_v_/F_m_ ratios (Figure 2).

### 2.3. Effects of High RZT on Productivity of RZ Heat Primed Plants and Non-Primed Plants

**Figure 3:**
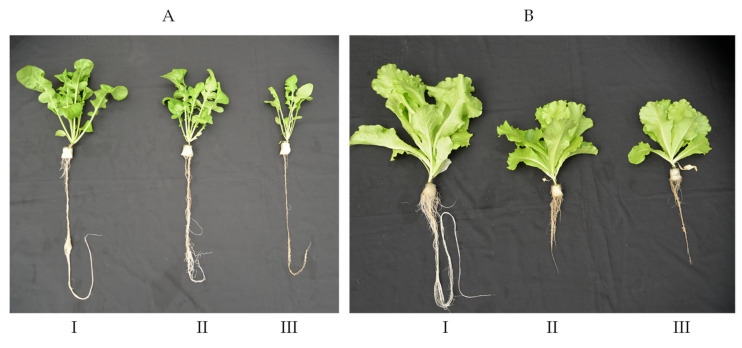
Arugula (**A**) and Canasta (**B**) plants grown under different RZTs at harvest (36 DAT). I, 25 °C-RZT; II, 25 °C-RZT → 38 °C-RZT → 42 °C-RZT (RZ heat primed plant) and III, 25 °C-RZT → 42 °C-RZT (non-primed plant). Refer to Section 4.2 for further details on different RZT treatments.

**Figure 4:**
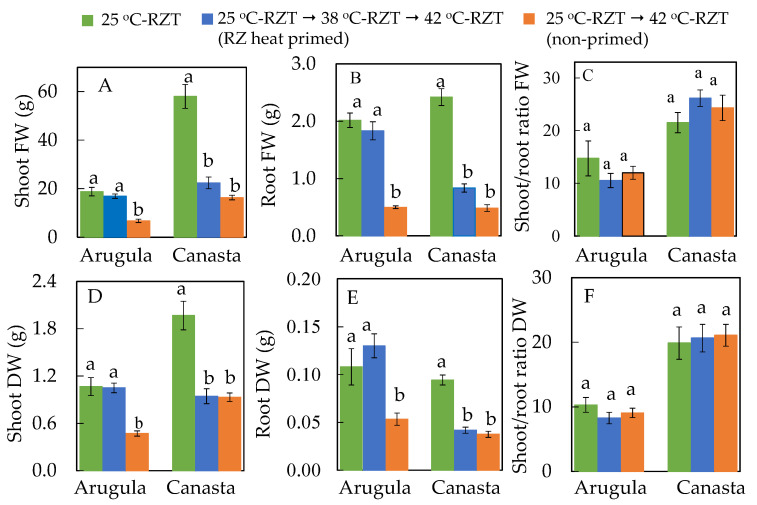
Shoot FW (**A**), root FW (**B**), shoot/root ratio FW (**C**), shoot DW (**D**), root DW (**E**), shoot/root ratio DW (**F**) at harvest (36 DAT). The vertical lines through the means represent standard errors (*n* = 4). Different lowercase letters are statistically different (*p* < 0.05, Tukey’s multiple comparison test). Each species was analyzed separately. Refer to Section 4.2 for further details on different RZT treatments.

Figure 3 shows Arugula (Figure 3A) and Canasta (Figure 3B) plants grown under different RZTs for 36 days. Although all plants look healthy, non-primed plants were smaller compared to those grown under 25 °C-RZT for both species. For Arugula, the size of RZ heat primed plants was much bigger than that of non-primed plants. This was supported by the fact that for shoot fresh weight (FW), root FW and dry weight (DW) (Figure 4A,B,D,E), there were no significant differences between 25 °C-RZT Arugula and RZ heat primed Arugula, which were significantly higher than that of non-primed Arugula plants. However, both RZ heat primed Canasta and non-primed Canasta had similarly smaller size with lower shoot and root FW and DW compared to that of 25 °C-RZT plants (Figure 3B and Figure 4A,B,D,E). All plants had similar shoot/root ratio FW (Figure 4C) and shoot/root ratio DW (Figure 4F).

### 2.4. Effects of High RZT on Root Morphology of RZ Heat Primed and Non-Primed Plants

**Figure 5:**
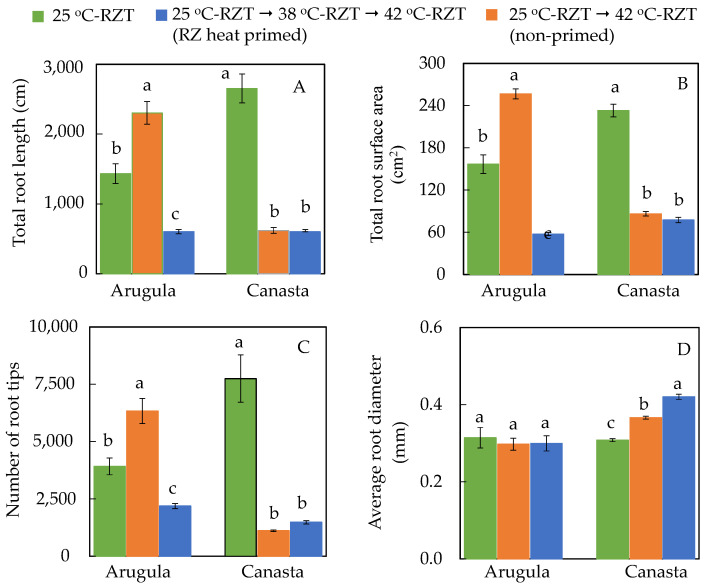
Total root length (**A**), total root surface area (**B**), total number of root tips (**C**) and average root diameter (**D**) of Arugula and Canasta at harvest (36 DAT). The vertical lines through the means represent standard errors (*n* = 4). Different lowercase letters are statistically different (*p* < 0.05, Tukey’s multiple comparison test). Each species was analyzed separately. Refer to Section 4.2 for further details on different RZT treatments.

For Arugula, RZ heat priming increased total root length (Figure 5A), total root surface area (Figure 5B) and total number of root tips (Figure 5C) compared to those of 25 °C-RZT and non-primed plants at harvest. However, all Arugula plants had similar average root diameter (Figure 5D). For both RZ heat primed and non-primed Canasta plants, there were no significance differences in total root length, surface area and number of root tip, but these parameters were significantly lower than those of 25 °C-RZT Canasta (Figure 5A–C). Moreover, there was significant difference in the average root diameter of Canasta across all treatments, with non-primed plants having significantly the highest root diameter; the lowest root diameter was observed in 25 °C-RZT plants (Figure 5D).

### 2.5. Nutritional Quality at Harvest and during Postharvest Storage of RZ Heat Primed and Non-Primed Plants after Subjecting to High RZT

**Figure 6:**
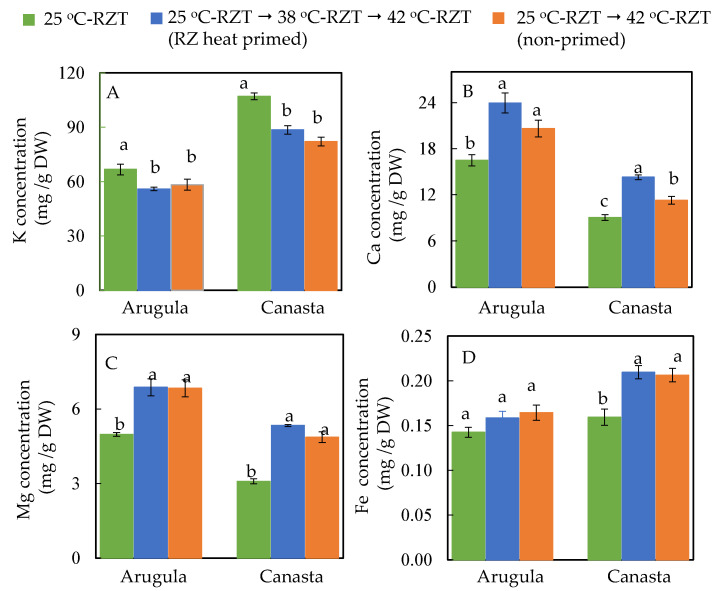
Concentrations of K (**A**), Ca (**B**), Mg (**C**) and Fe (**D**) of Arugula and Canasta at harvest (36 DAT). The vertical lines through the means represent standard errors (*n* = 4). Different lowercase letters are statistically different (*p* < 0.05, Tukey’s multiple comparison test). Each species was analyzed separately. Refer to Section 4.2 for further details on different RZT treatments.

Since the concentration of leaf dietary minerals does not vary much during postharvest storage [28], this study only took into account the concentrations of four important dietary minerals such as K, Ca, Mg and Fe at harvest. Both RZ heat primed and non-primed Arugula and Canasta had significantly higher Ca (Figure 6B) and Mg concentrations (Figure 6C) but significantly lower K concentration (Figure 6A) compared to those of 25 °C-RZT plants. For Fe concentration, all Arugula plants had similar concentration while Fe concentration of Canasta was significantly higher in RZ heat primed and non-primed plants than in 25 °C-RZT plants (Figure 6D).

**Figure 7:**
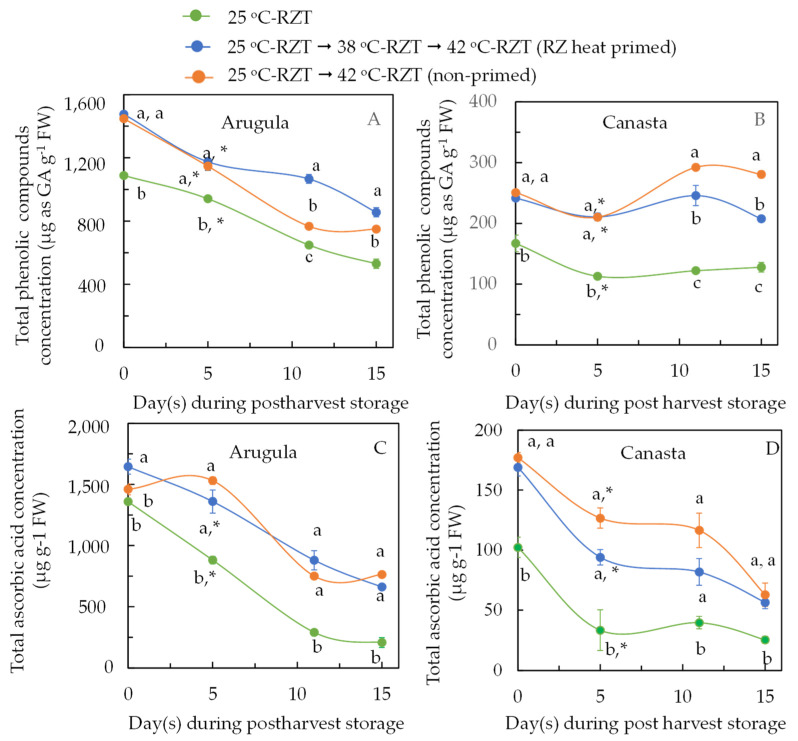
Total phenolic compounds concentration (**A**,**B**) and total ascorbic acid concentration (**C**,**D**) of Arugula (**A**,**C**) and Canasta (**B**,**D**) at harvest (36 DAT or 0 day after harvest) and during postharvest storage. The vertical lines through the means represent standard errors (*n* = 4). Different lowercase letters are statistically different (*p* < 0.05, Tukey’s multiple comparison test) among different RZT treatments. Samples from each postharvest day were analyzed separately. For the changes of total phenolic compounds and ascorbic acid between 0 day (at harvest) and 5 days after postharvest storage, means with an asterisk (*) indicate significantly different (*p* < 0.05) using Dunnett’s procedure. Refer to Section 4.2 for further details on different RZT treatments.

At harvest (0 day after harvest), the total phenolic compound concentrations in both Arugula (Figure 7A) and Canasta (Figure 7B) were significantly higher in RZ heat primed and non-primed plants than in 25 °C-RZT plants. For the total ascorbic acid concentration, RZ heat primed Arugula plants had the highest values while non-primed Arugula and 25 °C-RZT Arugula plants had similar lower values (Figure 7C). The total ascorbic acid concentration in Canasta (Figure 7D) was significantly higher in RZ heat primed and non-primed plants than in 25 °C-RZT plants. During postharvest storage, total phenolic compounds and total ascorbic acid concentrations generally decreased in Arugula as storage time increased (Figure 7A,C). As most people tend to store their vegetables for, at most, around 5 days, a comparison by Dunnett’s test for both total phenolic compounds and total ascorbic acid across all treatments between at harvest and 5 days after harvest was made. There was a significant decrease in total phenolic compound concentration for all Arugula and Canasta plants 5 days after postharvest storage except for total ascorbic acids in non-primed Arugula plants. However, at each given period, both RZ heat primed and non-primed Arugula plants had significantly higher total phenolic compounds and total ascorbic acid concentrations compared to those of 25 °C-RZT plants over the 15 days of postharvest storage period. Similar trends occurred for the total ascorbic acid concentration observed in different Canasta plants during postharvest storage period (Figure 7D). However, total phenolic compound concentration seemed to remain constant for all Canasta during postharvest storage or even slightly higher in non-primed Canasta plants (Figure 7B).

## 3. Discussion

In our previous studies, it was found that subtropical and temperate vegetable crops grown under hot A-RZT exhibited reduced maximum quantum efficiency of PSII measured by midday F_v_/F_m_ ratio from the dark-adapted leaves compared to those of the same species grown under cool-RZT of 20 °C or 25 °C [15,16,17,18]. In this study, midday F_v_/F_m_ ratios of Arugula and Canasta after RZ heat priming for 10 days decreased to approximately 0.6 (Figure 1A,B), which were significantly lower compared to those of 25 °C-RZT plants. This result shows that when both temperate vegetable crops were subjected to 38 °C-RZT during midday, dynamic photoinhibition had occurred due to the midday’s higher photosynthetic photon flux density (PPFD) and high leaf temperature in conjunction with the high RZT [17]. Dynamic photoinhibition in Canasta leaves during RZ heat priming leading to a decline in overall photosynthetic performance [17,29,30,31] could have resulted in its significantly lower leaf area expansion than 25 °C-RZT Canasta leaves (Figure 1D). The impact of RZ heat priming on leaf growth may be species-dependent since there was no statistical difference in the leaf area expansion of Arugula leaves (Figure 1C) despite its significantly lower midday F_v_/F_m_ ratio (Figure 1A). However, all plants had similar predawn F_v_/F_m_ ratios which were greater than 0.8 (data not shown). This finding suggests that all plants had fully recovered overnight and that no chronic photoinhibition occurred in any plants during RZ heat priming [30,31].

The abrupt exposure to severe heat stress of 42 °C-RZT could have resulted in significantly lower F_v_/F_m_ ratios for non-primed plants (25 °C-RZT → 42 °C-RZT plants), particularly the F_v_/F_m_ ratio of non-primed Arugula which reached close to 0.6 (Figure 2). This agrees with the findings of Lai and He [32] in which F_v_/F_m_ ratios of six temperate crops, including Arugula and Canasta, decreased with increasing heat stress. Although F_v_/F_m_ ratios of 25 °C-RZT Arugula leaves were the highest, there was no significant difference between the F_v_/F_m_ ratios of RZ heat primed (25 °C-RZT → 38 °C-RZT → 42 °C) and 25 °C-RZT Arugula leaves (Figure 2). This implies that RZ heat priming may have improved the thermotolerance of PSII for Arugula leaves, helping plants acclimatize to high temperatures [33]. However, improved thermotolerance was not observed in RZ heat primed Canasta as F_v_/F_m_ ratios of both RZ heat primed and non-primed Canasta were significantly lower after subjecting to 42 °C-RZT compared to those of 25 °C-RZT.

RZT plays a very important role in shoot development [15,16,19,34]. Results shown in Figure 3 and Figure 4 supported our previous studies conducted on temperate vegetable crops grown under high RZTs which had lower shoot and root FW and DW [15,16,19]. Through RZ heat priming, Arugula may have acquired thermotolerance, minimizing reduction in shoot and root productivity (Figure 3A and Figure 4A,B,D,E). This could be attributed to the altered root morphology of the RZ heat primed Arugula plants (Figure 5). After subjecting to high RZT of 42 °C during midday, RZ heat priming may have led to Arugula plants going through a faster rate of root growth and development when it is cooler during the night. This was reflected by the results of total root length (Figure 5A), total root surface area (Figure 5B) and total number of root tips (Figure 5C) which were, respectively, enhanced by 61%, 64% and 62% compared to those Arugula plants grown at 25 °C-RZT. Our previous studies on temperate lettuce [17,35] and Arugula [36] plants showed that extensive root systems grown under cool-RZTs increased transpiration cooling by facilitating the movement of water, moderating leaf temperature. Thus, RZ heat primed Arugula could have resorted to altering its root system as an acclimatization strategy. However, effects of RZ heat priming on root morphology depend on species. In the present study, both RZ heat primed and non-primed Canasta plants had significantly shorter total root length (Figure 5A), smaller root surface area (Figure 5B), fewer number of root tips (Figure 5C) but greater root diameter (Figure 5D). Poor developed root systems of RZ heat primed and non-primed Canasta could have restricted water and mineral uptake and thus reduced their root and shoot productivity, which was evident from their significant lower shoot and root FW and DW (Figure 4A,B,D,E). Despite the effects of RZT on root morphology and productivity, no significant difference was observed in the shoot/root ratio FW and DW (Figure 4C,F) for both species among different RZT treatments, suggesting that high RZT did not affect photoassimilate partitioning between shoot and root.

Root morphological traits contribute directly to water and mineral uptake [37]. RZT plays an important role in affecting root morphology as well as water and mineral nutrient uptake in temperate crops grown in tropical conditions [38]. In this study, plants were grown with aeroponic system which is a powerful tool for the study of RZT on root morphology and mineral nutrition [35,36,38,39]. Uptake of mineral nutrients relies on the distances and surface area to which the roots extend vertically and horizontally [38]. For instance, temperate lettuce grown at a RZT of 20 °C had a larger root system with longer length, larger surface area and with more tips compared to those grown at hot A-RZT. It was found that lettuce plants grown at 20 °C-RZT generally accumulated higher concentrations of both macro- and micro-nutrients than those of lettuce plants grown under hot A-RZT [38]. In this study, we mainly focused on the accumulations of major dietary minerals such as K, Ca, Mg and Fe. At harvest, compared to 25 °C-RZT plants, Ca, Mg and Fe (Figure 6B–D) concentrations were significantly higher in both RZ heat primed and non-primed plants of both species. Higher concentrations of Ca, Mg and Fe in RZ heat primed Arugula could be resulted from its larger root system (Figure 5A–C) whereas for non-primed Arugula, RZ heat primed and non-primed Canaster, high concentrations of minerals may be resulted from these minerals being concentrated within small plants (Figure 3) compared to those grown at 25 °C-RZT. However, both Arugula and Canasta plants grown under 25 °C-RZT had significantly higher K concentration than those RZ heat primed and non-primed plants (Figure 6A), indicating that the uptake of K was negative affected by high RZT [38].

Phenolic compounds are an essential class of secondary metabolites that help plants to be more tolerant to heat stress as they not only serve as a form of photoprotection but also reduce leaf osmotic potential, facilitating uptake of water [21]. The effects of RZT on the accumulation of secondary metabolites such as phenolic compounds in plants have been reported recently [40,41]. For instance, in the study with coriander (*Coriandrum sativum* L.), total phenolic concentrations were greatest under 30 °C-RZT compared to those grown under 20 °C-RZT and 25 °C-RZT [41]. This study also found that both RZ heat primed and non-primed Arugula (Figure 7A) and Canasta (Figure 7B) at harvest had significantly higher total phenolic compound concentrations after exposure to high RZT of 42 °C for 16 days compared to those grown in 25 °C-RZT plants. Heat stress may also induce oxidative stress, producing reactive oxygen species (ROS) such as hydrogen peroxide (H_2_O_2_). To prevent photo-oxidative damage, plants activate their ROS scavenger system which involves the synthesis of ascorbic acid, an antioxidant which confers resistance to abiotic stress to lower H_2_O_2_ concentration [21]. It was reported that the antioxidant capacity of the coriander (*C. sativum* L.) plant was greatest under combination of PPFD of 300 μmol m**^−^**^2^ s**^−^**^1^ and 30 °C-RZT compared to those under lower PPFD and lower RZT [42]. In this study, not only RZ heat primed but also non-primed Arugula (Figure 7C) and Canasta (Figure 7D) synthesized greater amounts of ascorbic acid to counteract the heat-induced oxidative stress after exposure to 42 °C-RZT in the late growth stage than those grown under 25 °C-RZT for the entire growth period.

Degradation of phytochemicals may have occurred during storage [42]. In this study, total phenolic compounds in Arugula (Figure 7A) and total ascorbic acid in Arugula (Figure 7C) and Canasta (Figure 7D) generally decreased during postharvest storage for a period of 15 days. As most of the working class tend to store their vegetables for 5 to 6 days, the change in total phenolic compounds and ascorbic acid from day 0 (at harvest) to day 5 after postharvest storage was highlighted in discussing the postharvest quality of vegetables. Based on the results of Dunnett’s test, except for total ascorbic acids in non-primed Arugula plants (Figure 7C), all plants had significantly lower total phenolic compounds and total ascorbic acid 5 days after postharvest storage compared to those at harvest for all plants (Figure 7A–C). However, both RZ heat primed and non-primed Arugula and Canasta plants had significantly higher total phenolic compounds and total ascorbic acid compared to those of 25 °C-RZT plants. These findings suggest that high RZT enhances postharvest quality [25,26].

## 4. Materials and Methods

### 4.1. Plant Materials

**Figure 8:**
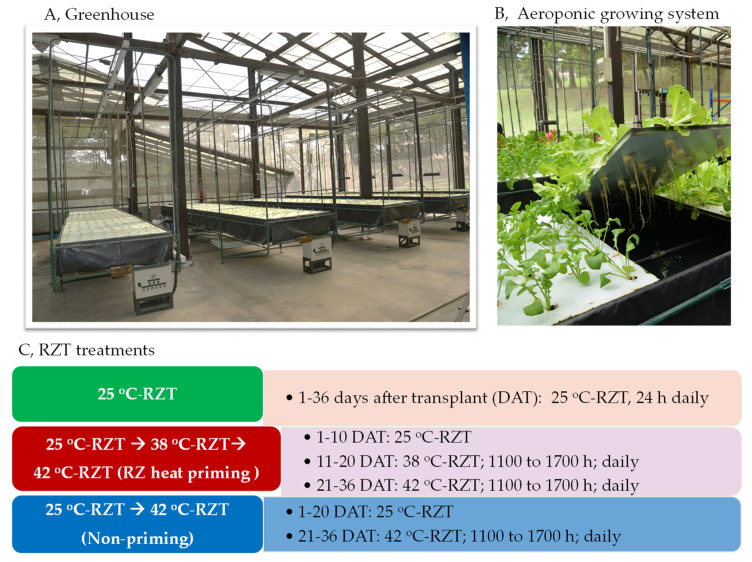
Photos of the tropical greenhouse (**A**) and aeroponic growing system (**B**) and different RZT treatments (**C**).

After 3 days of germination, seedlings of Arugula (*E. sativa*) and Canasta (*L. sativa*) were inserted into polyurethane cubes saturated with water and placed under sunlight for a week before transplanting into aeroponic systems in the tropical greenhouse (Figure 8A,B). The plant roots were exposed at 25 °C for 24 h daily in the trough but the shoots were grown under ambient temperatures. All plants were supplied with full-strength Netherlands Standard Composition nutrient solution in the form of spray mist for one minute at every 5-min interval. The composition of full-strength nutrient solution was described in our previous study [22]. The pH and electrical conductivity of the nutrient solution were maintained at 6.0 ± 0.2 and 2.0 ± 0.2 mS cm**^−^**^1^, respectively, throughout the growth period. The average maximal PPFD was about 600 μmol m**^−^**^2^ s**^−^**^1^.

### 4.2. RZT Treatments

The aerial parts of all plants were grown under diurnal fluctuating ambient temperatures (26 °C–40 °C) in the hot tropical greenhouse (Figure 8A,B). The RZ of all plants were subjected to the following treatments: (1) 25 °C-RZT: plants were kept at a constant 25 °C-RZT for 24 h daily for 36 DAT; (2) 25 °C-RZT → 38 °C-RZT → 42 °C-RZT (RZ heat priming): plants were grown at a constant 25 °C-RZT, 24 h daily for the first 10 days, then subjected to fluctuating hot A-RZT with a constant 38 °C-RZT maintained from 1100 to 1700 h for 6 h daily from 11 to 20 DAT and then exposed to fluctuating hot A-RZT with a constant 42 °C-RZT maintained for 16 days from 1100 to 1700 h for 6 h daily and (3) 25 °C-RZT → 42 °C-RZT (non-priming): plants were grown at a constant 25 °C-RZT 24 h daily for 20 days and for the last 16 days were transferred to fluctuating hot A-RZT with a constant 42 °C-RZT maintained from 1100 to 1700 h for 6 h daily (Figure 8C).

### 4.3. Postharvest Shoot Sampling and Storage

On 36 DAT, all harvested shoot samples from all treatments were stored in a 4 °C refrigerator in the same size of Ziploc bags. Four plants from each treatment of both species were successively removed after 0 (defined as at harvest), 5, 11 and 15 days of postharvest storage for nutritional quality analysis.

### 4.4. Measurement of F_v_/F_m_ Ratio

F_v_/F_m_ ratio were measured from the dark-adapted leaves (15 min in the darkness) during midday (1200 to 1300 h) using the Plant Efficiency Analyser (Hansatech Instruments Ltd., King’s Lynn, England). The initial fluorescence, F_o_, was recorded before assessing the maximum fluorescence, F_m_, by 0.8 s of saturated pulse (>6000 μmquol∙m**^−^**^2^ s**^−^**^1^). Variable fluorescence, F_v_, was calculated as F_v_ = F_m_ − F_o_.

### 4.5. Measurement of Leaf Area Expansion

Between 10 and 20 DAT, plants from each treatment were tagged and newly emerged leaves were identified for leaf expansion measurement. Images of leaves placed individually on a white background with a scale drawn were taken at 2-day intervals and were analyzed using ImageJ software (NIH, Bethesda, MD, USA) to determine the leaf area according to the method outlined by Schneider et al. [43].

### 4.6. Root Morphology Analysis

The WIN MAC RHIZO V 3.9 program was used for root morphology analysis on 36 DAT. For each plant, the roots were detached before being placed in a tray of water which spread the roots out. After scanning the roots with WIN MAC RHIZO scanner, the values of different root parameters were determined by the program.

### 4.7. Measurements of Shoot and Root Productivity

Plants were harvested between 1000 and 1200 h on 36 DAT. Shoots and roots were separated for FW measurement before they were wrapped in aluminum foil and dried at 80 °C for 4 days to obtain DW. The dried shoots were then kept for analysis of dietary minerals.

### 4.8. Measurements of Shoot Dietary Minerals

Dried samples (0.2 g) were digested in 4 mL of 65% nitric acid (UltraWAVE, Milestone, Shelton, CT, USA) before diluting with Milli-Q water (final volume: 25 mL). The Optima 8300 coupled plasma optical emission spectrophotometry (ICP-OES) and WinLab 32 (Perkin Elmer, Waltham, MA, USA) [44] were used to determine the concentrations of dietary minerals.

### 4.9. Determination of Total Phenolic Compounds

Fresh leaves (0.5 g) were ground in liquid nitrogen with 5 mL of 80% methanol [45]. The supernatants of extracts were used after shaking for 30 min at 200 rpm and centrifuged at 3500 rpm for 20 min. Before analyzing the total phenolic compounds, 0.5 mL of supernatant was diluted with 0.5 mL of Folin–Ciocalteau reagent and 1 mL of 7.5% sodium bicarbonate solution (Na_2_CO_3_). After 20 min, the absorbance at 765 nm was measured using a spectrophotometer (UV-2550 Shimadzu, Kyoto, Japan) [45].

### 4.10. Determination of Total Ascorbic Acid

Reduced and oxidized forms of ascorbic acid were assayed by the reduction of 2,6-dichlorophenolindophenol (DCPIP) [46]. Fresh shoots (0.5 g) were grounded with liquid nitrogen and NaCl (1 g) was added. Extraction was completed by adding 5 mL of ice-cold 2% (*w*/*v*) metaphosphoric acid. The homogenate was centrifuged at 9000 rpm for 30 min at 4 °C. An aliquot (0.3 mL) was mixed with 0.2 mL 45% (*w*/*v*) K2HPO4 and 0.1 mL 0.1% (*w*/*v*) homocysteine to reduce oxidized ascorbic acid before determining the total ascorbic acid content. After 15 min of incubation at 25 °C, 1 mL of 2 M citrate–phosphate buffer and 1 mL 0.003% (*w*/*v*) DCPIP were added. The absorbance at 524 nm was recorded using a spectrophotometer (UV-2550 Shimadzu, Kyoto, Japan) [46].

### 4.11. Statistical Analysis

SPSS statistics software was used to perform statistical analysis. Levene’s test was first carried out to ensure that variances across different RZT treatments and postharvest days were equal. One-way analysis of variances (ANOVA) and Tukey’s multiple comparison tests were used to discriminate among means of different RZT treatments, with *p* < 0.05 indicating that means were significantly different using different letters. For analysis of phytochemical results during postharvest storage, means obtained at harvest were assigned as the control and were compared with 5 days after postharvest storage using Dunnett’s procedure, with *p* < 0.05 indicating that means were significantly different from means obtained at harvest. Statistical analysis results obtained via Dunnett’s procedure were shown in Figure 7 with asterisks (*).

## 5. Conclusions

The results of this study have confirmed that sublethal RZ heat priming can induce thermotolerance in temperate leafy greens such as Arugula (*E. sativa*) plants but not Canasta (*L. sativa*). For Arugula, RZ heat priming mitigated the reduction of midday F_v_/F_m_ ratio, promoted root development, enhanced productivity and increased nutritional quality at harvest and during postharvest storage after subjecting to high RZT of 42 °C. For Canasta, although RZ heat priming also enhanced its nutritional quality after transferring them to 42 °C-RZT, its root development and productivity were negatively affected by high RZT.

## Data Availability

The data are available upon reasonable request in writing to the corresponding author.
